# Ocular Manifestations in Seborrheic Dermatitis Epidemiology, Clinical Features, and Management: A Comprehensive Review

**DOI:** 10.7759/cureus.70335

**Published:** 2024-09-27

**Authors:** Rasha M Alofi, Lujain S Alrohaily, Nada N Alharthi, May M Almouteri

**Affiliations:** 1 Family Medicine, Ministry of Health, Madinah, SAU; 2 College of Medicine, Taibah University, Madinah, SAU

**Keywords:** blepharitis, conjunctivitis, keratitis, ocular manifestations, seborrheic dermatitis

## Abstract

Seborrheic dermatitis (SD) often leads to ocular manifestations (OM) that are frequently overlooked. This study comprehensively explains the genesis of these ocular issues, which involves a combination of Malassezia overgrowth, changes in sebum production, and inflammatory responses in the body. The periocular region is rich in sebaceous glands, allowing Malassezia to thrive, which can lead to an inflammatory reaction that spreads to the eye surface, causing disorders such as blepharitis, conjunctivitis, keratitis, and ocular surface diseases. Although epidemiological data are limited, it is well established that ocular involvement occurs in approximately 10%-40% of individuals with SD. Early detection and treatment are crucial to prevent potential vision-threatening complications. A comprehensive diagnostic approach is necessary, including clinical examination, slit-lamp biomicroscopy, tear film analysis, and corneal imaging. Managing these conditions requires a multidisciplinary strategy involving collaboration between dermatologists and ophthalmologists. The treatment should involve topical and systemic medications to address the skin and ocular components. Patient education is critical for improving adherence to therapy, self-management, and the early identification of problems.

In the future, it will be essential to investigate the intricate interactions between Malassezia species and host immunological processes. This collective effort will involve creating new biomarkers and diagnostic tools, investigating targeted immunomodulatory drugs and novel lipid-based medicines as potential treatments, and conducting large-scale longitudinal studies to understand the epidemiological patterns and prognostic variables better. By raising awareness, encouraging collaboration across disciplines, and advancing research, healthcare practitioners can significantly improve patients' quality of life with SD and OM.

## Introduction and background

Seborrheic dermatitis (SD) is a persistent inflammatory skin disorder that primarily affects body regions and produces significant sebum, with the scalp, face, and trunk being the most affected areas [1]. Less than 1%-5% of the population is affected by SD [2,3]. Throughout infancy and adulthood, there are two peaks in the distribution of the SD incidence [4]. This distribution changed according to the age group. Foley et al. observed that the prevalence of SD in adults ranges from one to three percent, with the probability of males having SD being higher than that of females having SD [5]. Researchers have discovered several factors that contribute to the development of this condition, including Malassezia yeasts, hormonal effects, and immunological dysfunction [6]. However, the exact mechanism underlying its pathogenesis remains unclear.

The pathophysiology of ocular manifestations (OM) in SD is complex and poorly understood. Various processes have been linked to disease development. Malassezia yeasts, the regular occupants of the skin flora, have emerged as critical drivers of SD development [7]. These lipophilic microorganisms convert sebum into irritating fatty acids, producing an inflammatory cascade [8]. Patients with SD also exhibit immunological dysregulation, as indicated by increased proinflammatory cytokine production and activated T lymphocytes [9]. The interaction between skin microecology, lipid processing, and immune function underpins OM [9]. Ocular indicators of SD can vary significantly in severity and may damage various eye tissues, including the eyelids, conjunctiva, and cornea. Blepharitis, characterized by inflammation around the eyelid borders, is the most common ocular finding in patients with SD [10,11].

Quarterman et al. discovered that a substantial majority of SD patients (56%) had symptoms of blepharitis [12]. Conjunctivitis, keratitis, and dry eye syndrome have all been reported as possible ocular consequences of SD [13]. Arora et al. investigated OM in 100 people who were diagnosed with SD. The researchers found that ocular involvement was present in 68% of the patients, with blepharitis being the most prevalent illness (54%) and conjunctivitis being the second most common ailment (22%), followed by keratitis (8%). Moreover, this study demonstrated a significant association between the severity of SD and the occurrence of eye symptoms. More pronounced OM is possible in patients with substantial dermatological symptoms [13]. To successfully treat OM in Southeast Asian countries, an all-encompassing strategy that incorporates the knowledge and experience of dermatology and ophthalmology is required [13]. Treatment approaches are intended to be effective by addressing the underlying causes of skin problems, reducing inflammation, and relieving symptoms associated with the eyes [14]. Several studies have shown that topical antifungal medications such as ketoconazole and ciclopirox effectively treat SD [7].

While topical ketoconazole and pimecrolimus have shown efficacy for SD [15], caution is warranted when using these treatments near the eyes. Ketoconazole has been associated with ocular irritation in some cases [16], and the long-term ocular safety of pimecrolimus has not been extensively studied in the context of periocular use [17]. This review summarizes the existing information on ocular involvement in SD by reviewing its epidemiology, etiology, clinical characteristics, and therapeutic methods. Highlighting the visual features of SD and providing insights into its efficient management significantly improve comprehension. It is an essential resource for healthcare practitioners, guiding current clinical practice and future research.

## Review

Methods

Study Design and Data Sources

A comprehensive review procedure and online resources were utilized, including PubMed, Embase, and Web of Science. This process used a carefully created mix of pertinent keywords and topic titles linked to SD and associated OM. The reference lists were manually evaluated to ensure a comprehensive search. This was performed to identify any additional studies that could have been missed during computerized database searches.

Study Selection

Only articles from English-language journals subjected to peer review and primarily focused on the OM of SD were chosen for this study. To preserve rigorous evidence and minimize the likelihood of bias, editorials and opinion articles were not included in the investigation.

Methodological Rigor and Evidence-Based Approach

Due to the study's meticulous and methodological methodology, a comprehensive and evidence-based understanding of OM related to SD was achieved. A rigorous methodology, in conjunction with the diverse skills of the research team, boosted the study's capacity to produce information that is beneficial for clinical practice and contributes to future research efforts in this field.

Review

History

The history of SD and its OM spans over two centuries, with significant advancements in understanding its etiology, pathophysiology, and management. Robert Willan's pioneering description of OM in SD in 1808 marked a crucial milestone in recognizing the interconnection between skin and eye health [18,19]. This early observation lays the foundation for future research and a better clinical understanding. Stenson's subsequent focus on seborrheic blepharitis refined our knowledge of specific ocular involvement in SD. SD can cause discomfort and affect daily living because it is a recurring chronic condition [18-21].

Significant shifts in perspectives have marked the evolution of our understanding of SD and its OM. Early 19th-century European and Saxon ophthalmology schools have provided valuable insights into the ocular signs of SD. Compared to modern clinical and histological investigations, these historical accounts demonstrate how our comprehension of SD has progressed from purely descriptive observations to a more mechanistic understanding of the condition [19,22].

In 1808, Robert Willan made a groundbreaking contribution by describing the OM of the SD [23]. This study critically explains the relationship between ocular and cutaneous health. Stenson was the pioneer who investigated seborrheic blepharitis, a distinct form of eyelid inflammation that is linked to SD [24,25].

SD is frequently observed in clinics specializing in dermatology and ophthalmology [20,26]. Recent research has emphasized its relationship with various medical problems, stressing the importance of comprehensive examination and therapy for the illness [26,27]. As unexpected locations such as the external ear canal, pre-sternal and interscapular areas, axilla, and other moist or hairy regions have been discovered as possible manifestation sites, a holistic approach must be adopted [26,27]. Substantial investigations and documentation in the medical literature have revealed a connection between SD and ocular diseases, particularly seborrheic blepharitis [28,29]. This has helped healthcare professionals and researchers develop more effective therapeutic options. The involvement of eyebrows in Fuchs' dermatitis, a related disorder, has also been investigated, providing insight into this region's distinctive presentations and potential consequences [28,29].

Historical materials, such as ancient textbooks and reports from European and Saxon schools of ophthalmology from the 19th century, have drawn attention to the ocular signs of SD [20,30], providing significant insights into the nature of the condition. In later studies, researchers used clinical and histological methods to examine periocular involvement [1,31]. This allowed them to uncover the influence of allergens, allergic chemicals, and cancer on the conjunctiva of individuals with SD [20,31]. Several factors, including inheritance, hormonal imbalances, nutritional deficiencies, and environmental circumstances, can play a role in the development of SD, affecting people of any age or sex [20,32]. This condition frequently occurs when the immune system is compromised. It may be associated with other systemic conditions in certain instances, making its management and treatment more challenging [32,33]. Gray-white or straw-colored scales, hair loss, scalp thickness, and milder forms, such as dandruff, are symptoms that SD may cause. This condition primarily affects the scalp and can be embarrassing or uncomfortable [20,32,33].

Etiology

SD has a multifactorial etiology; it is caused by a combination of several variables that interact [34-36]. The primary theory regarding the cause of SD is the expansion of Malassezia species and commensal fungi found on the skin [33,34,36]. The pathogenesis of SD is influenced by multiple factors, including genetic predisposition, overactive sebaceous glands, excessive sebum production, neurogenic and neuroinflammatory variables, and disruption of the skin barrier function [36,37]. These factors can contribute to the development of the disorder by creating an environment favorable for the growth of the fungus Malassezia, leading to an inflammatory response. Additionally, dietary factors play a role in the development of SD [38,39].

Several possible factors have been identified, including excessive nutrition and vitamin B2 and B6 deficiency. The development of SD has been linked to several lifestyle and genetic variables, including, but not limited to, oily hair or skin products, stress, genetic vulnerability, harsh climatic circumstances, fear, physical fatigue, antibiotics, and oral contraceptives [39-41]. The periorbital area is abundant in sebaceous glands, creating a lipid-rich environment conducive to the expansion of Malassezia [40,41]. Owing to the close anatomical proximity between the skin and conjunctiva/cornea, it is easier for inflammatory processes to extend from the periocular area to the eye surface [42,43]. Therefore, ocular involvement is a common feature of SD [43].

Epidemiology

Recent epidemiological studies have shed light on the prevalence and distribution of SD. Haq et al. reported that SD affects up to 5% of the general population in the United States, with a higher incidence during infancy and middle age. When compared with earlier studies, these data suggest a potential increase in SD prevalence over time. However, it is crucial to consider that this apparent increase might be due to improved diagnostic criteria or heightened awareness among healthcare providers rather than an actual rise in incidence [44-46]. Several risk factors have been identified, including immunosuppression, neurological problems, and climatic conditions such as high humidity and temperature. Although no apparent sex preferences have been established, several risk factors have been identified [1,6,44,45]. Although the exact frequency of SD is not well-documented, OM is highly prevalent [1,6]. The prevalence of OM in SD patients varies across studies. While some research suggests that ocular involvement may occur in up to 37% % of patients [47,48], other studies report lower rates, ranging from 3% to 5% [1]. Discrepancies in study techniques, clinical diagnoses, and patient groups are likely responsible for the vast range of ocular involvement estimations. Despite this, it is evident that ocular problems are a significant expression of SD and deserve more attention and systematic assessment [1,6,49].

It is common for OM in SD to present with non-specific symptoms that may result in an incorrect diagnosis [13,50]. In some instances, the ocular form of the condition may be misdiagnosed as adenoviral ocular infection because of the similarities between the two illnesses [13,50]. This incorrect diagnosis can lead to inappropriate administration of ocular corticosteroids, resulting in adverse consequences [51,52]. Notably, the package inserts of these drugs do not provide any information on potential ocular reactions after administering multiple or continuous ocular corticosteroids. The primary treatment approach for SD with blepharitis consists of using antibiotic eye drops or ointments and methodical and consistent removal of exudate using a sponge [51,52]. As this symptom is widespread, increasing awareness and conducting more systematic examinations is essential to improve diagnostic accuracy, reduce misinterpretation, and ensure proper care [13,52-54].

Pathophysiology

SD is a common form of recurring chronic dermatitis, characterized by highly itchy and red skin and flaky or oily scales [54,55]. This condition is most observed in places with abundant sebum, such as the scalp, face, nasolabial folds, retro auricular region, sternum, and intertriginous regions, including the axillary folds, inguinal areas, and inframammary folds [46,54-56]. Owing to the complex interactions among sebum polarization, the presence of the fungus *Malassezia *spp., and genetic and environmental variables that influence immune responses, microbes, and skin barriers, the exact pathophysiology of SD remains difficult to comprehend [55,57,58].

Regarding the immune response, SD is characterized by inflammation induced by overactive immune reactions to Malassezia species that inhabit areas rich in sebum [6,58]. However, it is essential to recognize that genetic and environmental variables may also play a significant role in the pathophysiology of the illness. These factors can alter immunological functions in the surroundings of the sebum [6,58,59]. While the role of Malassezia species in SD is well-established [36], its specific involvement in OM, particularly seborrheic blepharitis, requires further investigation. Malassezia has been isolated from the eyelids of both healthy individuals and those with blepharitis [60], but its causal role in ocular disease is not yet conclusively proven.

The pathophysiologies of SD and OM are complex and multifactorial. Recent research has significantly advanced our understanding of the role of Malassezia yeast in SD development. Li et al. demonstrated a strong correlation between the presence of Malassezia hyphae and SD pathogenesis [37]. This finding builds upon an earlier work by Gupta and Bluhm [7], who identified Malassezia as a critical driver of SD. The interplay among Malassezia, sebum production, and host immune responses creates a complex pathogenic environment. Further studies are required to fully understand the complex nature of SD pathogenesis, with a particular focus on immune response mechanisms and the interaction between hereditary and environmental variables. Recognizing and appreciating these processes is essential to better understanding the elements related to SD and designing targeted treatments and interventions [6,55].

It is of utmost importance to investigate the relationship between surroundings that are abundant in sebum and immunological responses, as well as the influence of lifestyle variables and external triggers, such as stress and climatic conditions, on the symptoms of SD. Additionally, research should concentrate on discovering biomarkers and genetic variables that may contribute to an individual's vulnerability to SD and creating diagnostic methods that do not require any intrusive procedures.

Clinical Presentation

Ocular involvement in SD can occur in various ways, ranging from moderate irritation of the eyelids to severe illness of the ocular surface [6,59]. The most prevalent clinical signs of SD are ocular symptoms, ranging from moderate eyelid pain to severe ocular surface infection. Vital clinical characteristics include blepharitis, which involves inflammation of the eyelid margins characterized by crusting, scaling, and redness; conjunctivitis, characterized by redness, irritation, and discharge; meibomian gland dysfunction, resulting in dry eye symptoms due to obstruction and malfunction of the meibomian glands; keratitis, causing corneal inflammation that may impair vision; and ocular rosacea, which presents with OM similar to the severity of OM and varies greatly, and in some instances, ocular symptoms may arise before the development of cutaneous problems [6,61]. SD is a persistent and relapsing inflammatory skin disorder that often manifests in body regions that contain high concentrations of sebaceous glands, such as the scalp, face, and external ear canal [43,59,61]. The most noticeable and clinically significant symptom is excessive dandruff scaling, which can be ugly, incredibly irritating, and unpleasant for the individual. It is commonly known that several factors can cause the condition to become more severe. These factors include stress, seasonal changes, and Malassezia [6,43,61].

When a disease affects the skin surrounding the eyelids, eyebrows, eyelashes, or the skin of the external ear canal, specific ocular and dermatological issues must be considered. The condition, known as SD, can exhibit itself in various ways, including in the eyes, and can cause many symptoms [43,61].

Significant lash inflammation, which leads to trichiasis, is an essential ocular ailment that has been seen. This can cause considerable discomfort and interfere with an individual’s ability to see [43,62]. Additionally, the creation of abscesses might result from Zeiss gland infestation, further complicating the disease. The widespread crusted inflammatory lid illness is another concerning pattern that can lead to significant eye irritation and inflammation. This could be the result of the disease [43,61,62].

In cases where a patient presents with scalp or facial SD along with constitutional symptoms or severe eye involvement, medical experts must undertake a comprehensive history and physical examination to evaluate the likelihood of underlying systemic disorders. To achieve effective outcomes in individuals with SD, prompt intervention and adequate therapy of both dermatological and ocular elements are crucial [43,56,61,62].

Physical Examination

Ocular problems are frequently reported by people with SD; however, there is ongoing debate as to whether these symptoms are directly related to seborrhea or whether they are suggestive of a distinct disorder known as atopic keratoconjunctivitis [63,64]. People with this condition frequently have symptoms such as burning sensations, itching, discomfort, impaired vision, photosensitivity, and a sense of having something alien in their eyes [63,64]. A wide spectrum of eye symptoms can occur, ranging from minor to severe symptoms. In less severe instances, individuals may not experience any symptoms or may only occasionally experience moderate itching [64,65]. In contrast, a significant number of people have reported experiencing itching, which interferes with their ability to sleep, particularly early in the morning and late at night [66,67]. A “pins and needles” feeling, excessive tearing, and heightened sensitivity to light are usually present in conjunction with the minor pain that you are experiencing. In approximately 12.5% of cases, patients described feeling as though they had something alien in their eyes [64,67].

When a person is under stress, experiencing emotional strain, consuming alcohol, or consuming spicy foods, the symptoms frequently become more severe [13]. OM that is persistent and recurrent, such as redness or itching, is prevalent and probably not only associated with ocular seborrhea [13,67]. During ophthalmological examinations, doctors may observe lesions and infiltrates at the border of the eyelid, superficial scars, and telangiectatic blood vessels in severe cases. In addition, superficial scars may manifest. To diagnose ocular SD, it is necessary to investigate additional risk factors for meibomian gland dysfunction and anterior blepharitis respectively [13,67,68].

Several factors can lead to the development of ocular seborrhea. These factors include fluctuations in hormone levels; the use of cosmetics; deficiencies or imbalances in vitamins related to keratinization and lubrication; and external physical, biological, or chemical factors such as temperature, humidity, hydrogen peroxide, and the utilization of low-viscosity silicone respiratory masks [13,67,68]. Individuals who are vulnerable to these variables may develop irritation, surface infections caused by bacteria or fungi, or seborrhea of the hair follicles or surrounding regions. The presence of fungal yeasts and their byproducts on the scalp surface can worsen dandruff symptoms, including increased skin shedding, pain, and inflammation. Dandruff affects the scalp [13,67]. Although all patients with scalp seborrhea also suffer from OM at the same time, the precise association between these clinical manifestations and seborrhea remains unknown [13,69]. Topical corticosteroids, which are the conventional therapy for dandruff, only provide brief relief, and standard solutions are typically ineffective and may even be detrimental to the eye surfaces [13,64,70].

Dermatologists need to focus on thorough and specialized care when treating ocular SD. To obtain the best possible results, ocular surface treatment should be initiated before administering corticosteroids [70-72]. To enhance the quality of life of affected individuals, it is essential to adopt a multifaceted strategy to address the underlying causes and provide relief from symptoms [71,72]. Supporting patients in the management and reduction of OM associated with SD can be accomplished by medical professionals using holistic and individualized therapeutic approaches [64,69-71].

Evaluation

In individuals with SD, it is essential to examine ocular involvement to facilitate timely diagnosis and management of any potential problems [73,74]. To facilitate the early diagnosis of OM, eye specialists recommend that all patients diagnosed with SD undergo annual ophthalmic evaluations [73-75]. In situations in which OM is evident, such as blepharitis or conjunctivitis, it is necessary to assess the ophthalmologist as soon as possible [74,76]. This is of utmost significance because there is a possibility of major involvement of the anterior segment and immediate intervention is required to avoid consequences [76]. If one believes that they may have ocular SD, they must seek advice from eye care specialists and undergo an ophthalmological examination [74,76].

When such situations are observed, an ophthalmologist should conduct a thorough systemic physical examination, with particular emphasis on the orbital and ocular regions of the body. Several common problems require quick eye assessment [74,76]. Symptoms include ocular discomfort, redness, foreign bodies, itching, and inflammation. In addition, it is vital to assess facial skin problems in patients with a history of blepharitis. Additionally, it is essential to undertake a comprehensive tarsal checkup for patients with blepharitis and meibomian gland dysfunction [64,76]. Patients may also present with symptoms, such as vision abnormalities, light sensitivity, changes in visual acuity, and ocular discomfort or modifications due to SD-mediated keratitis. In such cases, it is imperative that patients undergo ophthalmological examination as soon as possible [64,74].

A complete ophthalmological examination is required to diagnose ocular problems associated with SD. This examination should include an evaluation of the patient's visual acuity, slit-lamp biomicroscopy, and, if necessary, diagnostic procedures, such as tear film analysis and corneal imaging [76]. Distinctive symptoms of inflammation and disturbance of the ocular surface can be identified by careful evaluation of eyelid margins, conjunctiva, and cornea. Objective measurements of tear film quality, such as tear breakup time and Schirmer's test, can be utilized to gain a better understanding of the severity of dry eye illness. Additionally, in vivo, confocal microscopy and impression cytology are two methods that may be used to assess cellular and structural alterations that occur inside the surface of the eye [76].

In 1997, Weldon et al. were the first to describe diagnostic criteria for ocular SD. These criteria included the presence of eye inflammation caused by SD, the presence of SD near the affected eye, the absence of any other causes of eye inflammation, and improvement in symptoms following the application of topical corticosteroids, desquamating agents, and dandruff lotion. When attempting to differentiate ocular SD from other conditions such as corticosteroid-induced eyelid dermatitis, atopic dermatitis, tinea of the eyelid, squamous cell carcinoma of the eyelid, rosacea of the eyelid, and conjunctivitis, it is essential to consider the history of the disease, presence of specific findings, and involvement of other areas [64,76]. The ability of healthcare providers to offer appropriate care and ensure the overall well-being of patients with SD and its possible OM is contingent upon their adherence to the approved diagnostic criteria and their ability to conduct a full ophthalmological examination [64,76].

Management

SD therapy entails a multidisciplinary strategy with the goals of lowering inflammation; treating symptoms such as itching, redness, and peeling; and preventing future recurrences of the condition [64,77]. The ideal technique is determined by the severity of the condition and the area of the affected body. This includes considering whether the afflicted area is located in areas that are hair-bearing or have specialized skin [64,76]. It is essential to keep in mind that SD therapy is typically a preventative, long-term strategy rather than a curative one, even though it can effectively control the signs and symptoms for longer periods of time [64,76,77]. Treatment options for SD are presented in Table 1.

**Table 1:** Treatment options for SD SD: Seborrheic dermatitis

Treatment category	Function
Corticosteroids	Alleviate irritation and itching.
Azole antifungals	Target the yeast responsible for the ailment.
Calcineurin inhibitors	Regulate immunological responses to reduce symptoms.
Keratolytic agents (salicylic acid, coal tar derivatives)	Assist in the elimination of scales and reduce irritation by promoting the shedding of skin cells.

In addition to being employed topically, these medications can be incorporated into shampoos or scalp treatments. For more severe instances, systemic treatment may be considered [64,77]. Over-the-counter medicated shampoos and scalp treatments that include active ingredients such as ketoconazole, zinc pyrithione, and selenium sulfide are growing in popularity as therapeutic alternatives for individuals with mild to moderate SD. To achieve optimal outcomes, it is crucial to follow usage instructions carefully [76,77]. In addition to the use of medicated products, the use of personalized hair care products, avoidance of dietary factors that can exacerbate the condition (such as excessive amounts of sugar or dairy), and maintaining excellent overall skin hygiene, including regular washing and moisturization with appropriate products, can effectively manage symptoms and reduce the risk of flare-ups [64,78].

The treatment of ocular problems related to SD often involves a multimodal approach that addresses both the underlying skin conditions and symptoms observed in the eyes [77-79]. The most common treatment methods for eyelid and conjunctival involvement include lid hygiene, warm compresses, and topical anti-inflammatory medications (corticosteroids or calcineurin inhibitors) [64,74]. In cases where the condition is severe or treatment-resistant, systemic therapy, such as oral antifungal or anti-inflammatory drugs, may be tried [76,77]. If corneal issues arise, it may be necessary to use topical corticosteroids, antibacterial eye drops, and artificial tears without preservatives to reduce inflammation and promote healing [77,78]. Meibomian gland dysfunction may require the use of specific therapies, such as heat treatment, lid massage, and eye drops that include lipids [64,78].

In cases requiring coordination between dermatologists and ophthalmologists, it is crucial to establish effective collaboration to deliver comprehensive care and achieve optimal patient outcomes [77,79]. Patient age and the location of the condition may influence the recommended treatment approach [64,78]. For infants with cradle caps, gentle shampooing, and brushing are recommended, while various scalp treatments, including medicated shampoos and preparations containing steroids, coal tar, salicylic acid, or selenium sulfide, may be utilized for hair-bearing areas, such as the scalp and beard, that experience mild-to-moderate symptoms [13,78]. It is essential to consult a healthcare expert, such as a dermatologist or ophthalmologist, for an accurate diagnosis, a tailored treatment plan, regular progress monitoring, and optimal outcomes [64,76,78].

To ensure the best possible outcomes for patients, dermatologists and ophthalmologists must collaborate closely in cases involving skin conditions affecting the eyes [77,79]. The age of the patient and the location of the condition may influence the recommended treatment approach [64,78]. For infants with cradle caps, gentle shampooing, and brushing are recommended, while various scalp treatments, including medicated shampoos and preparations containing steroids, coal tar, salicylic acid, or selenium sulfide, may be used for hair-bearing areas, such as the scalp and beard, that experience mild-to-moderate symptoms [13,78]. It is crucial to consult a healthcare expert, such as a dermatologist or ophthalmologist, for an accurate diagnosis, a tailored treatment plan, regular progress monitoring, and optimal outcomes [64,76,78].

Differential Diagnosis

As some cases may show symptoms that are not accurate, the clinical differential diagnosis of seborrheic blepharitis and its associated OM can be difficult [13,52,80]. This is because some patients may present with deceptive symptoms that could lead to treatment failure. It is crucial to consider other conditions that may be similar to SD, even if the diagnosis is often clear [13,24,80]. Similar to SD, other skin regions can be affected by common dermatological disorders. These conditions often manifest as greasy or scaling plaques, erythema, and pruritus as their characteristic symptoms [13,81]. In contrast, inflammatory skin disorders can localize to the margins of the eyelids in individuals initially examined for ocular infections caused by other factors [81,82]. Seborrheic blepharitis and its associated OM require careful consideration in the differential diagnosis. Various conditions must be considered, such as atopic and allergic contact; contact irritant blepharitis; dry eye syndrome; infectious dermatitis or blepharitis extending to the eyelids; and malignancies, including melanoma, basal cell carcinoma, or squamous cell carcinoma, particularly in cases of ulcerated, tumor-like, or lichenified eyelid lesions. Additionally, other eye disorders that may present similarly to seborrheic blepharitis include various forms of blepharitis (infectious, posterior, or mixed, e.g., staphylococcal or meibomian gland dysfunction), conjunctivitis (allergic, viral, bacterial), dry eye disease, ocular rosacea, atopic keratoconjunctivitis, meibomian gland dysfunction complications, contact lens-related complications, and infectious keratitis. Clinical and laboratory recommendations have been presented to aid in differential diagnosis, including a diagnostic checklist that suggests recommendations for empirical therapy of blepharitis. The checklist also considers various entities that may be implicated in the condition [82,83]. The present body of literature and the clinical consensus of dermatologists who practice ophthalmology serve as the basis for the development of these guidelines [13,79,84].

To discriminate between these entities and arrive at an appropriate diagnosis, it is necessary to conduct a thorough clinical examination, obtain a comprehensive patient history, and conduct specific diagnostic tests [83,85]. Close communication between ophthalmologists and dermatologists is essential for conducting a complete examination and developing individualized treatment plans [84,86,87]. In patients who have undergone breast augmentation, it is essential to consider phenomena such as naughty pupillary constriction, following phenylephrine administration. This phenomenon can assist in the identification of mechanical issues, such as ankyloblepharon, and address aesthetic concerns, such as the descent of the superior pole and asymmetry [13,83,86]. It is possible for medical practitioners to establish an accurate diagnosis and offer the best possible care for patients who exhibit OM associated with SD or other underlying disorders if they consider the numerous differential diagnoses and make use of relevant clinical and laboratory examinations [13,83,85].

Prognosis

Several different regions of the skin can be impacted by SD, which can be either persistent or recurring. This disease affects both the glabrous skin on the scalp and body, as well as the ocular region [3,88]. The severity of the illness, as well as the presence of any additional comorbidities, can have a significant effect on the prognosis of schizophrenia, which can vary considerably [3,32,89]. SD is often a persistent and recurrent disorder that lingers for an extended period and continues to manifest even after it appears to have been resolved [32]. The severity of scaly dermatitis can range from mild dandruff on the scalp to severe plaques on the face, scalp, or body, including inflammation of the body folds and ocular surfaces [3,27,90]. Patients with other conditions that frequently co-occur with SD may have a worse prognosis [27,32,91]. This is because they face additional challenges in managing their condition, which significantly impacts their quality of life [27,87,89]. People with SD may experience a decline in their quality of life due to physical, mental, and social stressors caused by the visible and distressing symptoms associated with the illness [32,92]. Schizophrenia is a chronic condition that requires long-term care and treatment, and it is characterized by relapses [43,93].

The long-term use of an appropriate skin care regimen is usually necessary even after the initial flare-up of symptoms has been adequately treated with suitable medication [94,95]. The use of shampoos on a daily basis is recommended for treating the scalp [90,96]. While scleroderma can reduce the rate at which certain substances are released from the scalp microenvironment, it does not completely stop their release, which may lead to a worsening of the symptoms associated with the condition [91,94,97]. Furthermore, research has shown that individuals with schizophrenia are more likely to be concerned about their skin and feel more stigmatized than those with other conditions such as hypertension, diabetes, and heart disease [92,98]. The visible signs of scleroderma, including redness, scaling, and skin lesions, can have a negative impact on patients' self-esteem and cause them to feel self-conscious [97,98]. This highlights the need for a holistic approach to managing scleroderma [97,98]. As such, it is essential to develop a comprehensive treatment strategy to effectively manage the condition. This strategy should go beyond simply reducing symptoms during flare-ups [27,98]. Maintaining the general health of the skin and mental and social well-being of individuals with scleroderma requires treating the underlying causes [27,99]. Healthcare providers can enhance the quality of life of those living with schizophrenia by gaining knowledge of the complex nature of the disorder and implementing solutions that can manage both the physical and emotional aspects of the condition. This approach would also provide patients with a more positive prognosis [27,93,98].

Complication

SD is a disorder that warrants serious attention, as it can lead to consequences that threaten vision [25,74]. The persistent inflammation and scarring that occur inside the peripheral parts of the anterior region of the eye [13,100,101] are the root causes of these issues. Although treatments are available to alleviate symptoms, these options frequently fail to halt the progression of the underlying pathophysiology and growth associated with this disorder [74,102,103]. Instead of solely focusing on symptom management, it is crucial to address and treat the underlying cause of SD, which is SD within the anterior region. Neglecting to do so could have severe consequences, including permanent vision loss [13,25]. Persistent ocular symptoms can lead to several complications, such as chronic blepharitis and meibomian gland dysfunction, resulting in chronic dry eye disease, recurrent conjunctivitis, and keratitis, with the risk of vision-threatening corneal involvement; cicatricial changes like ectropion or entropion due to chronic eyelid inflammation; increased susceptibility to secondary bacterial or fungal infections; and diminished visual acuity and quality of life due to the cumulative effects of ocular surface disease.

The development of asymmetrical upper or lower eyelid retraction resulting from hyperemia of the tissues surrounding the eye is indicative of SD, a condition that is not afforded the same level of attention as other symptoms of the disorder [13,78,104]. Other consequences that have been observed include sudden quadrantanopsia resulting from chalazia, localized inflammation of the lacrimal gland, the formation of zonular cataracts in individuals with otherwise healthy immune systems, and anterior uveitis of varying causes [104-106]. Regrettably, individuals afflicted with this ailment often fail to inform their dermatologists or ophthalmologists about their eye-related symptoms or consequences, resulting in a more severe condition [3,100,105]. A lack of participation on the part of the patient can lead to a misunderstanding of the disease's progression or the effectiveness of the chosen treatment [34,57,103]. To minimize the likelihood of complications and achieve optimal long-term results, it is crucial to provide patients with comprehensive information, adhere to the recommended treatment regimen, and schedule regular follow-up appointments with dermatologists and ophthalmologists [4,100]. To assess the course of the disease, it is essential to investigate various treatment options and consider the multidimensional aspects of the problem [13,54,100].

Deterrence and Patient Education

Patient education is an essential component of the treatment of ocular problems associated with SD [102,103,106]. Patients must be provided with the necessary knowledge to make informed decisions and to actively participate in the management of ocular problems related to SD. To achieve this, it is crucial to educate patients about the pathophysiology of the condition and the role of Malassezia in initiating an inflammatory response. Additionally, proper lid hygiene techniques, including warm compression, gentle lid cleansers, and appropriate eyelid massage methods should be taught. It is important to provide information on various treatment options, including topical and systemic therapies, and their potential side effects. Regular follow-up appointments with dermatologists and ophthalmologists are also essential, and patients should be encouraged to promptly report any worsening eye symptoms or new indicators. They should also be advised to wash their hair regularly with shampoos that do not meet their eyes. The use of antifungals and steroid eye drops can be beneficial, but patients should be cautioned about their off-label use. Follow-up examinations using validated scales, such as the SD Severity Index (SDSP), and other optometric tests are necessary.

Patients should be informed about the potential risks associated with certain procedures, such as cataract formation and glaucoma, especially for those with preexisting conditions. It is also crucial to educate patients about the risks of prolonged sun exposure and the importance of protecting their eyes from harmful ultraviolet rays and high-energy visible light. Furthermore, patients with chronic side effects should seek advice from a dermatologist to undergo patch testing and investigate other treatment alternatives that are appropriate for their situation [100,102,104].

Implications for Clinical Practice

To manage ocular problems associated with SD, a multidisciplinary strategy involving close coordination between dermatologists and ophthalmologists is required [102,107]. Recommendations for managing the ocular signs of SD are presented in Table 2.

**Table 2:** Recommendations for managing ocular signs of SD SD: Seborrheic dermatitis; OM: ocular manifestations

Recommendation	Details
Increased awareness	Primary care clinicians, dermatologists, and ophthalmologists should recognize ocular signs of SD: redness, itching, burning, excessive tears, light sensitivity, and impaired vision.
Routine screening and comprehensive evaluation	Patients with SD should receive routine ophthalmological assessments to detect eye involvement, even in the absence of obvious OM.
Utilization of diagnostic tools	Employ objective diagnostic methods like tear film analysis and ocular surface imaging to accurately assess the extent and severity of ocular involvement.
Targeted treatment strategies	Implement treatment regimens that include both topical and systemic medications to address both cutaneous and ocular symptoms.
Collaboration and communication	Establish clear communication and referral pathways between dermatologists and ophthalmologists to ensure coordinated care.
Patient education and empowerment	Provide ongoing education and empower patients for improved adherence to therapy and effective self-management, enhancing overall outcomes.

Moreover, it is essential to differentiate SD from other facial disorders that may be mistaken for it to prevent unnecessary treatment [106,108]. Several variables, including the presence of androgen receptor-laden cells in the sebum glands and the possible impact of the skin microbiome, have been proposed as potential contributors to SD. However, the underlying pathophysiology and origin of this condition are not entirely comprehended [100,107,108]. In some cases, the use of systemic medication, in addition to topical therapies, should be considered, particularly when ocular signs are significant. Individuals suspected of having SD should be closely monitored, particularly for the presence of OM [102,109].

Future Directions in Research

The advancement of our knowledge of SD and the ocular issues associated with it has led to the identification of several promising avenues for future research. These include investigating the role of different Malassezia species in triggering ocular inflammatory responses and their interactions with the host immune system, developing new biomarkers and diagnostic techniques to better describe the severity and progression of ocular involvement, exploring innovative therapeutic approaches such as targeted immunomodulatory drugs and lipid-based treatments, evaluating long-term outcomes and potential preventive care options, and conducting large-scale longitudinal studies to elucidate epidemiological patterns, risk factors, and prognostic variables. These research directions aim to improve our understanding of SD-related ocular issues, enhance diagnostic capabilities, develop more effective treatments, and inform preventive strategies, ultimately leading to better management and outcomes for patients with SD-associated ocular problems. Moreover, researchers and doctors must continue their efforts to gain a deeper understanding of the pathophysiology of SD and its long-term consequences associated with the condition [107]. Although many environmental and genetic risk factors have been identified, their specific functions are not yet fully understood. Additionally, the development of sustainable and effective therapeutic interventions is essential, as currently available treatments only provide temporary relief from symptoms and recurrence is a common occurrence [56,77,108].

Research in the field of dermatology has made significant strides, as evidenced by the substantial advancements in our comprehension of the intrinsic skin microbiome and the variations in the microbiome between individuals [105,106]. It has been determined that most skin disorders, including SD, are attributable to the buildup of excessive amounts of the fungus Malassezia and its subsequent activation [103,107,110]. By gaining a deeper understanding of the intricate relationship between the skin microbiome and various skin conditions, scientists can revolutionize the treatment and management of these illnesses [56,109,111]. By addressing these research goals and utilizing recent breakthroughs in dermatological research, the scientific community can contribute to a more comprehensive understanding of SD, enhance its diagnostic capabilities, and develop more effective and targeted treatment approaches [7,110,111]. Ultimately, these efforts will improve the management of this complex and demanding disease, resulting in better patient outcomes and overall quality of life [27,109,111].

## Conclusions

SD primarily a dermatological disorder affecting sebum-rich regions like the scalp, face, and trunk, can lead to considerable and sometimes overlooked ocular consequences. These consequences include blepharitis, conjunctivitis, keratitis, and ocular surface diseases, which can potentially affect vision and the overall health of the eyes. This study emphasizes the multifactorial pathogenesis underlying ocular signs. This pathogenesis entails interactions between Malassezia overgrowth, sebum production, and host inflammatory responses. It is crucial to perform a comprehensive diagnostic workup, including clinical examination, slit-lamp biomicroscopy, tear film analysis, and corneal imaging. To achieve this, a multidisciplinary strategy is required for effective care by dermatologists and ophthalmologists working together. The treatment should incorporate topical and systemic medications to simultaneously address the cutaneous and ocular aspects of the condition. One of the most significant benefits of patient education is that it encourages treatment adherence, self-management, and early complication diagnosis. In the future, critical research tasks include clarifying the roles of Malassezia and host immunity, creating innovative biomarkers and diagnostic tools, assessing emerging therapies, and conducting large-scale epidemiological investigations. Ultimately, healthcare providers can significantly improve the complete treatment and outcomes of patients with SD and OM by increasing awareness, promoting multidisciplinary teamwork, and driving future research.
